# Supplementary material for: The Construction and Exploration of a Comprehensive MicroRNA Centered Regulatory Network in Foxtail Millet (Setaria italica L.)
Source: Front Plant Sci. 2022 May 6;13:848474. doi: 10.3389/fpls.2022.848474 (PMC9121102; doi:10.3389/fpls.2022.848474)
Supplement: Supplementary file 2 [file Data_Sheet_2.zip › Sit-miRN3023b_Seita.2G320900.1_6256_TPlot.pdf]

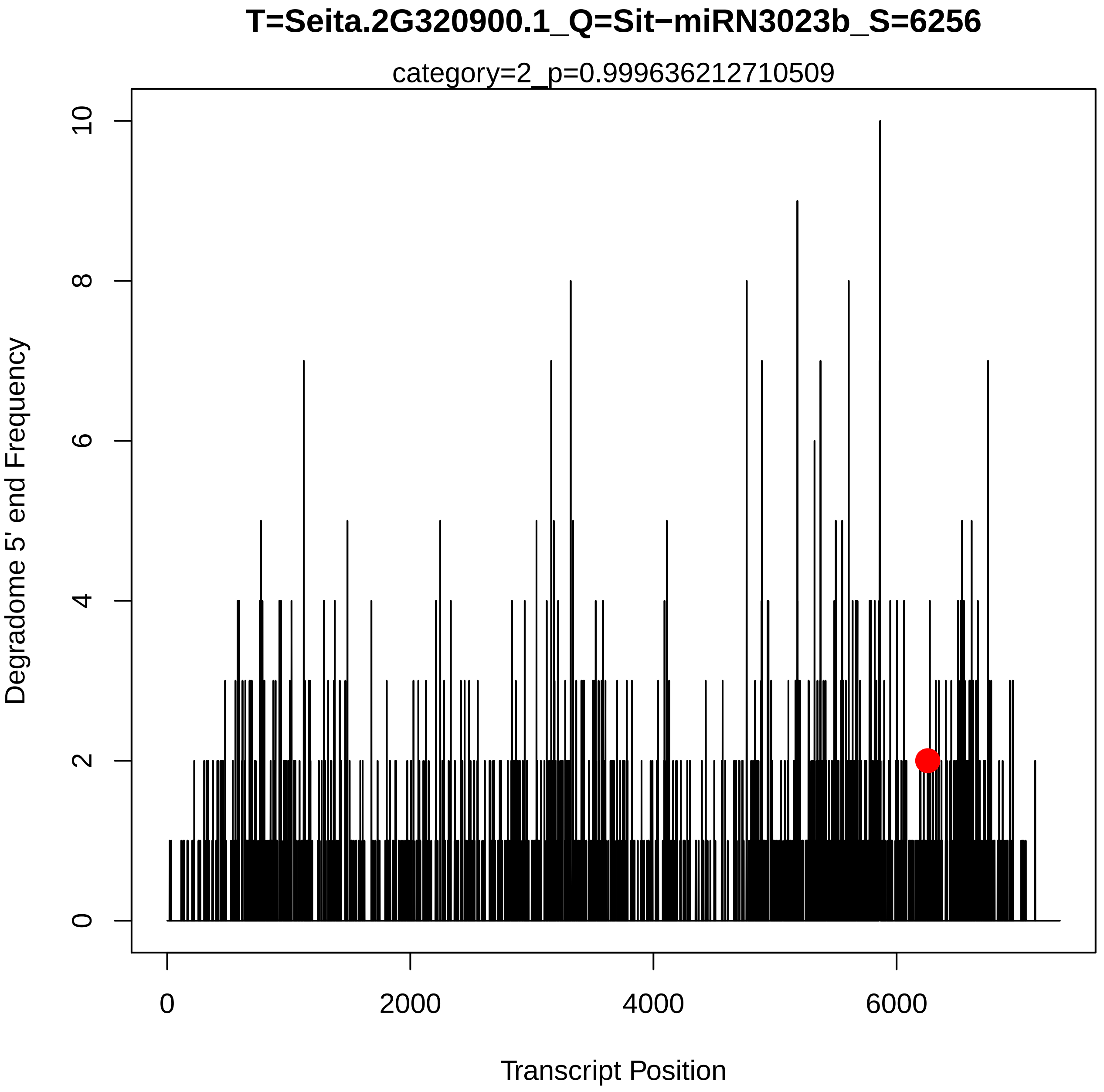
T=Seita.2G320900.1_Q=Sit−miRN3023b_S=6256
category=2_p=0.999636212710509
Degradome 5' end Frequency
0
2
4
6
8
10
0
2000
4000
6000
Transcript Position
